# Efficacy of intrauterine insemination in women with endometrioma-associated subfertility: analysis using propensity score matching

**DOI:** 10.1186/s12884-021-04342-y

**Published:** 2022-01-04

**Authors:** He Cai, Jinlin Xie, Juanzi Shi, Hui Wang

**Affiliations:** Assisted Reproduction Center, Northwest Women’s and Children’s Hospital, Houzaimen North Street, 73# Xi’an, People’s Republic of China

**Keywords:** Intrauterine insemination, Endometrioma, Subfertility, Propensity score matching

## Abstract

**Background:**

Intrauterine insemination (IUI) treatment is recommended in subfertile women with AFS/ASRM stage I/II endometriosis. However, the efficacy of IUI in women with ovarian endometriomas with tubal patency is uncertain. We explored the efficacy of IUI for the treatment of endometrioma-associated subfertility.

**Methods:**

We performed a retrospective matched cohort study using propensity matching (PSM) analysis. Subfertile couples undergoing IUI with and without ovarian stimulation between January 1, 2015, and May 30, 2020 were reviewed.

**Results:**

After PSM, 56 women with endometrioma alone were matched to 173 patients with unexplained subfertility. The per-cycle pregnancy rate (PR) was comparable between women with endometrioma-associated subfertility (*n* = 56, 87 cycles) and women with unexplained subfertility (*n* = 173, 280 cycles) (9.2% vs. 17.9%, OR 0.47; 95% CI, 0.21–1.03). Subgroup analyses based on IUI with or without stimulation also resulted in comparable results. A trend toward a lower cumulative pregnancy rates (CPRs) was seen in women with endometrioma (14.3%, 8/56) compared with women with unexplained subfertility (28.9%, 50/173), but the differences were not significant (HR 0.49; 95% CI, 0.23–1.15). However, patients with endometrioma were nearly twice as likely to converse to IVF treatment compared with those without the disease (60.7% versus 43.9%; OR 1.97; 95% CI, 1.07–3.65).

**Conclusion:**

IUI may be a viable approach for subfertile women with endometrioma and no other identifiable infertility factor. More studies are needed to reassure the findings.

**Supplementary Information:**

The online version contains supplementary material available at 10.1186/s12884-021-04342-y.

## Background

Endometriosis is one of the most common chronic gynecologic disorders and is frequently associated with female subfertility (up to 50% subfertile women with endometriosis) [1–3]. Approximately 190 million women worldwide are currently affected by endometriosis [4] and 30% to 50% of women with endometriosis are infertile [5–7]. Assisted reproductive technology (ART) frequently is used as the first-line therapy to endometriosis-associated infertility [8–10]. Guidelines of both the European Society of Human Reproduction and Embryology [9] and American Society for Reproductive Medicine [10], state that intrauterine insemination (IUI) treatment is only recommended in subfertile women with minimal-to-mild endometriosis. Werbrouck et al. reported no difference in cycle pregnancy rate between women with surgically treated minimal to mild endometriosis and women with unexplained infertility after controlled ovarian hyperstimulation and IUI program. The cumulative live-birth rate within four cycles of IUI was also comparable in women with minimal endometriosis, mild endometriosis, and unexplained infertility (70.2%, 68.2%, 66.5%, respectively) [11].

Ovarian endometriomas are found in 17%‐44% of women with endometriosis [12–14]. Although the exact pathophysiology of the reduced fertility is not clear, toxic content from an endometrioma may play a crucial role. Endometrioma is also usually overlap with those a more advanced stage of disease (stages III and IV of endometriosis according to the American Society for Reproductive Medicine (ASRM) classification).

A significant number of women with endometrioma will eventually seek ART to achieve a pregnancy, which is more often in those with reduced ovarian reserve or other identifiable infertility factor. However, for subfertile patients with presence of endometrioma alone (i.e.normal ovarian reserve and patency of fallopian tubes), is IUI treatment effective for subfertility associated with endometrioma? The optimal management often poses a clinical debate and little evidence exists to provide robust guidance to clinicians.

Considering a sequence of IUI cycles is less aggressive and less expensive than an IVF procedure, should IUI treatment be encouraged as a valuable or viable approach to achieve a natural pregnancy? The aim of the present study was to test the efficacy of IUI treatment on women with endometrioma-associated subfertility, comparing the fertility outcomes (per-cycle-pregnancy rate [PR], and cumulative pregnancy rates [CPRs] after IUI treatment in subfertile women with endometrioma and women with unexplained subfertility by using propensity matching (PSM) analysis.

## Methods

### Study design

We retrospectively analyzed the IUI with the husband’s sperm cycles performed from January 1, 2015 to May 30, 2020. The data were extracted from the database of infertility center of Northwest Women and Children’s Hospital, Xi’an, China. This study was approved by the institutional research ethics review board (2,019,013).

### Patients

Before reproductive treatment, all subfertile couples underwent a infertility evaluation, including physical examination, transvaginal ultrasound, cycle day 2–3 serum follicle-stimulating hormone (FSH) and luteinizing hormone (LH) assays, hysterosalpingography, semen analyses and associated safety tests.

The study group consisted of all consecutive subfertile women with the presence of identified endometrioma and no other identifiable infertility factor. Inclusing criteria were failure to conceive for ≥ 12 months, female age ≤ 40 years, ovulation demonstrated by appropriately timed mid-luteal progesterone,bilateral tubal patency (demonstrated by hysterosalpingography or laparoscopy); normal semen variables (according to World Health Organization criteria) [15]. Patients satisfied the above criteria were included whether or not the they had prior surgical treatment for their endometrioma.The endometrioma was diagnosed either by laparoscopy or a combination of physical examination and transvaginal ultrasound.

The control group consisted of couples diagnosed with subfertility who were treated during the same period of time and who met the same inclusion criteria except with no evidence of endometrioma. Women were excluded from the study if they had achieved a clinical pregnancy during the previous IUI treatment. Cases from IVF with controlled ovarian stimulation conversion to IUI due to low ovarian response were also excluded.The study and control groups of patients were matched using PSM analysis.

### IUI procedures and semen preparation

IUI was performed in natural or stimulated cycles [16]. Briefly, for natural cycles, the ultrasound and serum hormone tests started on the eighth day of the cycle. When the leading follicle was ≥ 14 mm, patients started the test for urinary LH; For stimulation cycles, ovarian stimulation was conducted by either administering letrozole or hMG (Menotropins for Injection, Livzon pharmaceutical group INC., China) or letrozole plus hMG. Ovarian response was monitored by the follicular growth and serum E_2_ levels starting on day 5 of stimulation, and then dose of hMG was adjusted accordingly every 1–3 days. IUI was performed 24 h after detection of LH in the urine. If the leading follicle measured over 18 mm in diameter in the absence of LH in the urine, 10,000 IU of hCG were administrated and insemination was performed the next day.

Semen was prepared on the day of insemination by centrifugation on a density gradientas, as previously described [17]. All women were treated by the same two physicians (HW and JLX) with the same IUI procedure.The prepared sperm was gently inserted within 1 cm of the fundal extend of the uterine cavity using a soft catheter. Micronized progesterone (200 mg/day) was used for 15 days after IUI.

### Outcome Measures

A serum β-hCG test was performed approximately 16 days after insemination. A clinical pregnancy was diagnosed 2 weeks after a positive test by the presence of a gestational sac on ultrasound. Live birth was defined as a live-born delivery at least 24 weeks after IUI. The primary outcomes of interest were clinical pregnancy rate (PR) per cycle and cumulative pregnancy rates (CPRs) after IUI treatment. A subanalysis was performed based on IUI protocols: natural or stimulation cycle.

### Statistical analysis

PSM was performed to adjust for confounding factors correlated with pregnancy outcomes. The variables in the PSM included female characteristics (i.e., age, gravidity, parity, body mass index (BMI), duration of infertility, antral follicle count (AFC), serum concentrations of FSH and LH, and male characteristics (age, semen parameters). To optimize the precision of the study, patients with endometrioma were matched to patients with unexplained infertility in a 1:3 matching ratio. The PSM allowed each endometrioma patient undergoing IUI to be matched to a unexplained infertile patient with similar characteristics.

PR-per cycle was compared between the two groups (endometrioma-associated subfertility vs. unexplained subfertility). A subanalysis was performed based on IUI protocols: natural or stimulation cycle. As one couple could have more IUI cycles we applied generalized estimating equations (GEE) that took into account this clustering. CPRs were the number of women who achieve a clinical pregnancy after one, two, or three added cycles divided by the number of women who started treatment.

Data were expressed as mean ± standard deviation (SD) or n (%). Descriptive data were compared by Student’s T, Mann–Whitney U, Chi-squared or Fishers’ exact tests when appropriate. The number of cycles since first IUI treatment were used as time parameters. The date of entry was the date of the first IUI treatment cycle. Patients were followed up to 1 year after finishing their last IUI treatment. Cox regression, adjusted for maternal age, was used to compare CPRs. Statistical analysis was analyzed using R (v.3.4.3; The R Foundation). *P* < 0.05 was considered to be significant.

Sensitivity analyses were performed on the ovarian endometrioma group versus the unexplained subfertility group with exclusion of patients without surgical diagnosis of endometrioma prior to IUI treatment.

## Results

### Patient characteristics

Based on inclusion criteria, 58 women with endometrioma and 880 women with unexplained infertility were available for analysis. After PSM, a total of 56 women with endometrioma were successfully matched to 173 women with unexplained infertility. No differences in age (female and male), BMI, duration of infertility, gravity and parity, AFC, basal FSH, LH levels or sperm parameters were found between the two matched groups after matching. Clinical and biological characteristics of patients before and after PSM were shown in Table 1. For women in the endometrioma group, 22 cases (39.3%) had undergone prior surgery for endometriomas before IUI treatment and the remaining 34 (60.7%) were diagnosed based on clinical and ultrasound evaluation.

**Table 1:** Patient characteristics before and after propensity score matching (PSM)

	Before PSM		*P*	After PSM		*P*
	Endometrioma-associated subfertility (*n* = 58)	Unexplained subfertility (*n* = 880)		Endometrioma-associated subfertility (*n* = 56)	Unexplained subfertility (*n* = 173)	
Maternal age (years)	30.78 (4.02)	30.22 (3.71)	0.272	30.46 (3.72)	30.08 (3.38)	0.466
Paternal age (years)	32.43 (4.67)	31.79 (4.36)	0.283	31.98 (4.10)	31.45 (4.26)	0.409
BMI (kg/m^2^)	21.86 (2.83)	22.00 (3.21)	0.755	21.85 (2.88)	21.48 (2.77)	0.403
Subfertility time (months)	32.38 (20.26)	33.12 (17.40)	0.756	32.89 (20.40)	32.46 (16.01)	0.833
Primary infertility (%)	40 (69.0)	554 (63.0)	0.358	39 (69.6)	119 (68.8)	0.904
Nulliparity (%)	53 (91.4)	765 (93.5)	0.326	52 (92.9)	158 (91.3)	0.719
AFC (n)	10.00 (4.78)	12.75 (5.58)	< 0.001	10.21 (4.72)	10.87 (3.79)	0.293
Day-3 FSH (mIU/mL)	7.31 (1.79)	7.04 (1.75)	0.250	7.31 (1.82)	7.32 (1.60)	0.973
Day-3 LH (mIU/mL)	4.39 (1.56)	4.62 (2.29)	0.460	4.40 (1.58)	4.71 (3.26)	0.485
Semen concentration (10^6^/mL)	62.54 (27.01)	62.93 (27.27)	0.916	62.78 (27.36)	62.12 (26.03)	0.872
Semen motility (%)	54.26 (13.77)	57.54 (13.18)	0.067	54.44 (13.93)	54.90 (12.32)	0.815
Normal semen morphology (%)	5.64 (1.66)	5.48 (1.70)	0.493	5.70 (1.66)	5.54 (1.93)	0.594

### Treatment outcomes

The 56 women from endometrioma group underwent a total of 87 cycles of IUI (range 1–4, a mean of 1.46 attempts per patient), including 45 natural cycles and 42 cycles with stimulation. While the 173 women with unexplained infertility underwent a total of 280 cycles of IUI (range 1–5, a mean of 1.51 attempts per patient), including 152 natural cycles and 128 cycles with stimulation.

### PR per-cycle

The per-cycle clinical PR was lower in women with endometrioma (*n* = 56, 87 cycles) than in the women with unexplained infertility (*n* = 173, 280 cycles), though this was of borderline statistical significance (9.2% vs. 17.9%, OR 0.47; 95% CI, 0.21–1.03, *p* = 0.06). The subgroup analyses based on IUI with or without stimulation also resulted in comparable results (Table 2).

**Table 2:** Pregnancy rates in per IUI cycle treatment

	Endometrioma- associated subfertility (*n* = 56)	Unexplained subfertility (*n* = 173)	OR (95% CI)	*P*-value
IUI cycles, n	87	280		
PR, n (%)	8 (9.2)	50 (17.9)	0.47 (0.21–1.03)	0.058
Subgroups				
Natural cycles, n	45	152		0.095
PR, n (%)	3 (6.7)	26 (17.1)	0.35 (0.10–1.20)	
Stimulation cycles, n	42	128		
PR, n (%)	5 (11.9)	24 (18.8)	0.59 (0.21–1.65)	0.310

*IUI* intrauterine Insemination, *PR* Pregnancy rate

The *P*-value is calculated using GEE taking into account repeated measurements per individual, to correct for the fact that individuals were allowed to participate more than once in this study (see Statistical analysis section)

When compared with natural cycles, IUI with stimulation cycles seemed to result in a slightly higher PR per cycle in the group with endometriomas (11.9% vs. 6.7%, *p* = 0.40), though the differences were not significant. No significant difference between the two strategies was observed in the subset of unexplained subfertility (18.8% vs. 17.1%, *p* = 0.72).

### CPRs

In the whole population, the overall CPRs was 15.7% after one IUI cycle, 23.6% after two, 24.9% after three and 25.3% after the final attempt. The specific CPRs at the first IUI cycle in the endometrioma group compared with unexplained infertility group were, respectively, 8.7% versus 18.5%; at the second cycle, 14.3% versus 32.4%; For women with endometrioma-associated subfertility, all pregnancies occurred within the first-two cycles of IUI program. For women with unexplained infertility, three women conceived at the third attempt and one case conceived at the fifth cycle.

**Fig. 1:**
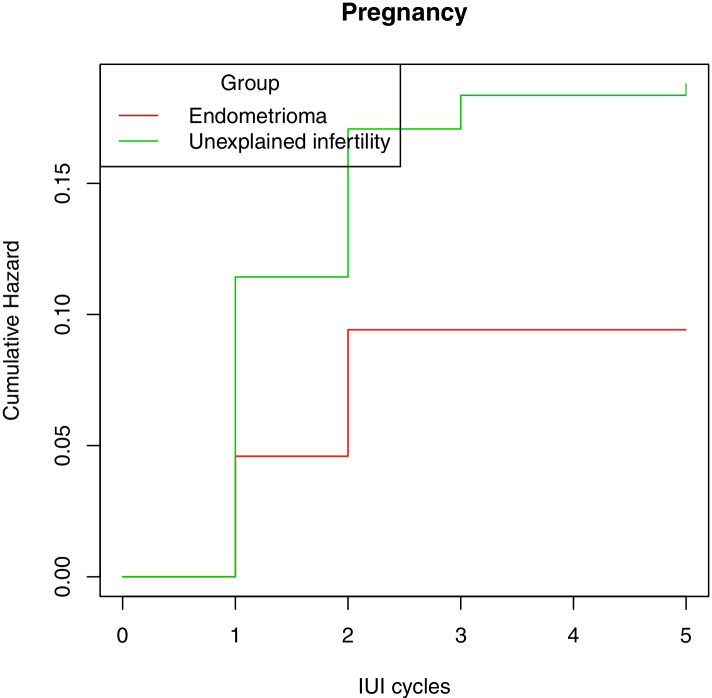
Cumulative pregnancy rates after IUI treatment in subfertile women of the two groups (Cox regression adjusted for maternal age)

The CPRs in women with endometrioma-associated subfertility (14.3%, 8/56) were comparable when compared with women with unexplained subfertility (28.9%, 50/173), (HR, 0.49; 95% CI, 0.23–1.15, *p* = 0.10) (Fig. 1). Women with endometrioma, however, were nearly twice as likely to converse to IVF treatment compared with those without the disease (60.7% versus 43.9%, respectively; OR, 1.97; 95% CI, 1.07–3.65). Among women with endometriomas, there were no differences in the size and number of unilateral or bilateral endometriomas between women who conceived and those not (see Additional file 1). Women who had surgical removal prior to IUI had similar CPRs compared with those women with no surgical treatment (3/22 or 13.6% versus 5/34 or 14.7%).

### Sensitivity analyses

The sensitivity analyses excluding women without surgically confirmed endometrioma (*n* = 34) also resulted in comparable results. The CPRs remained slightly lower in women with cystectomy prior starting IUI treatment (3/22; 13.6%) compared to the unexplained infertility women(50/173; 28.9%), but the difference was not significant. The number of pregnancies in each subgroup or combination, however, was small.

## Discussion

### Main findings

To our knowledge, this is the first report of a study focusing on the efficacy of IUI program in women with endometrioma-associated subfertility using a PSM technique. Compared with unexplained subfertile women in the matched group, the odds of per-cycle PR in subfertile women with endometriomas were 0.47 (95% CI, 0.21–1.03) and odds of CPRs were 0.49 (95% CI, 0.23–1.15). However, none of the estimations were statistically significant. We found that patients with endometriomas were nearly twice as likely to converse to IVF treatment compared with those without the disease. Subgroup analyses based on with/without prior surgery for endometrioma did not impact the outcomes. Although for women with endometrioma, stimulation cycles seemed to result in a slightly higher PR per cycle than that in natural cycles (11.9% vs. 6.7%, *p* = 0.40), the difference was not significant.

### Comparison to other studies

Similar findings have been reported for moderate-to-severe endometriosis. van der Houwen et al. [18] suggested that IUI was a valuable infertility management in women with more severe endometriosis, namely moderate-to-severe endometriosis. The CPRs of 28% in patients with Stage III and Stage IV endometriosis after six subsequent IUI cycles were reported. In the current study, the CPRs in women with endometrioma were lower than that in women with unexplained infertility (14.3% versus 28.9%; *p* = 0.10). There was a trend in favor of the unexplained infertility, but the number was probably too low to enable one to observe statistically significant differences from this aspect. It has been noted that majority couples in the study received no more than 4 cycles of IUI treatment. Although it reflects daily practice, we cannot exclude the possibility that exposure to more cycles of IUI could have led to a significant difference in CPR in both groups. In IUI cycles, women with endometrioma itself and its surgical resection may have adverse pregnancy outcomes compared to those without. Poor growing follicles, low oocyte quality have been described in women with endometriosis, all of which may potentially affect pregnancy outcomes [19]. In the present study, for women with endometrioma-associated subfertility, all pregnancies occurred within the first-two cycles of IUI program. Based on the finding, IUI may be effective in increasing probability of pregnancy in women with endometriomas, however, clinicians may consider performing IUI less than three cycles if they are not pregnant.

The optimum cycles of IUI attempts has been a pragmatic and challenge question when counseling couples. One previous publishing, concluded that IUI for treatment of unexplained infertility should be limited to a maximum of three cycles [20]. In the current study, a significant more women with endometrioma resorted to IVF treatment compared with those without the disease. We also found that for women with endometrioma, starting from the third cycle onward, additional attempts have only rarely increased fecundability. Although these could be attributed by chance, the results also suggested that women with subfertility and endometrioma may be reassured by more active and aggressive reproductive technology. we assume this information will be helpful in the counselling process. Some selected patients may be better served by IVF procedure if they fail to conceive after two cycles of IUI. Further data on this issue are needed.

It is still debatable whether IUI with stimulation is superior to unstimulated IUI. A pragmatic randomised controlled trial failed to show any advantage of superovulation over unstimulated IUI in couples with unexplained infertility [21]. While the Cochrane view supported clomifene citrate administration and suggested it had a beneficial effect in unexplained infertility [22]. In a recent study, an increased cumulative pregnancy rate has been shown in patients receiving IUI with stimulation up to six cycles compared to three times IUI without stimulation followed by up to three times IUI with stimulation, which endorsed the cochrane view [18]. In our study, when compared to IUI without stimulation, ovarian stimulation seemed to result in a slightly higher PR per cycle in women with subfertility and endometrioma (11.9% vs. 6.7%, *p* = 0.40). Differences in the two protocols that are close to statistical significance, increasing the sample size of patients may reveal meaningful role of ovarian stimulation in IUI program. The rationale for ovarian stimulation in women with endometrioma has been to correct potential disorders of endocrine and ovulation, including luteinized unruptured follicle syndrome, abnormal follicular growth, and premature LH surges [23].

However, ovarian stimulation may cause some concerns among patients. Impact of ovarian stimulation on the progression of endometriosis or its recurrence was recently summarized in a systematic review [24]. According to their report, impact of ovarian stimulation on ovarian endometrioma, if present, is clinically unremarkable. In the present study with multiple simulated IUI cycles, no bleeding, infection and other related complications was reported. All these results can be used to reassure patients.

### Strengths and limitations

One of the major strengths of this study was the use of PSM analysis to achieve matched groups. Evaluating and comparing treatment strategies for subfertile women with endometrioma alone is limited by heterogeneous practices between clinicians and centers. We specifically focused on this point by matching multiple clinical covariates in patients performing IUI. Secondly, at the present study, we compared the effectiveness of IUI on women with endometrioma alone versus women with unexplained subfertility. The assessment was specifically confined to the impact of ovarian endometrioma alone. Definition of the “true” unexplained infertility is still controversial. Some women in the unexplained subfertility group might have undetected minimal or mild endometriosis. However, that inclusion of the control group means that our results reflect the true contributory effect of the endometrioma alone with.

This study had some limitations deserve to be underlined. The relatively small sample size of the present study, may be underpowered to detect a significant difference in reproductive outcomes. Hence the results should be interpreted with caution. Our choice of clinical protocols for the management of subfertile women with endometrioma reflects current practice in our center and the rest of the China, but the results might not be generalizable to other populations and alternative national funding strategy. The inclusion of women with endometrioma could be diagnosed by laparoscopy or imaging detection might have introduced an factor of heterogeneity. Not reliably imaging peritoneal implants of endometriosis, however, transvaginal ultrasound have shown to have good accuracy for ovarian endometioma (95.1% ~ 96% specificity and 93 ~ 94.7% sensitivity) [25, 26], which is also recommended by ARSM [10]. There were few pregnancies within each subgroup (endometrioma diagnosed with/without prior surgery), sensitivity analyses indicate similar IUI treatment effects. We did not find any RCTs comparing reproductive outcomes after endometrioma cystectomy versus no treatment in women with endometrioma and addressing if IUI procedure is more successful post cystectomy compared to untreated. Future research should focus on more uniform control group and addressing the issues.

## Conclusion

The choice of the best treatment for endometrioma-assocaited subfertility remains a challenge. In the current study, IUI treatment may be a viable option to achieve spontaneous pregnancy for endometrioma subfertility. These findings must be confirmed by further studies. Individualized treatment based on the patient's age, clinical condition, costs and insurances is is highlighted.

## Supplementary Information

**Additional file 1.** Clinical parameters in women with endometrioma-associated subfertility.

## Data Availability

Data available on request corresponding author due to privacy and ethical restrictions.

## Abbreviations

AFC: Antral follicle count
ART: Assisted reproductive technology
ASRM: American Society for Reproductive Medicine
CPR: Cumulative pregnancy rates
FSH: Follicle-stimulating hormone
GEE: Generalized estimating equations
IUI: Intrauterine insemination
LH: Luteinizing hormone
PSM: Propensity score matching
PR: Pregnancy rate
