# Supplementary material for: Efficacy of intrauterine insemination in women with endometrioma-associated subfertility: analysis using propensity score matching
Source: BMC Pregnancy Childbirth. 2022 Jan 4;22:12. doi: 10.1186/s12884-021-04342-y (PMC8725563; doi:10.1186/s12884-021-04342-y)
Supplement: Supplementary file 1 — Additional file 1. Clinical parameters in women with endometrioma-associated subfertility. [file 12884_2021_4342_MOESM1_ESM.docx]

Additional file 1. Clinical parameters in women with endometrioma-associated subfertility

|  | Total | Pregnancy | Non-pregnancy | *p*-value |
| --- | --- | --- | --- | --- |
| Number, n | 56 | 8 | 48 |  |
| Size of the endometriomas  Mean±SD (range),mm | 28.7±9.8  (10-59) | 24.7±7.6  (15-35) | 28.9±10.1  (10-59) | 0.27 |
| Endometrioma location |  |  |  |  |
| Unilateral, n (%) | 47(83.9) | 7 (87.5) | 40 (83.3) | 0.77 |
